# Association of Breakfast Food Types with Dietary Knowledge, Attitudes, and Practices Among School-Aged Children

**DOI:** 10.3390/nu17152424

**Published:** 2025-07-24

**Authors:** Siyao Zhou, Hanqing Zhao, Yu Xiao, Jie Li, Qiaoli Huang, Yufang Zhang, Fengfeng Guo, Beibei Xu, Haoyan Zou, Xiaoxia Huang, Sizhe Huang, Lijun Wang

**Affiliations:** 1 Department of Nutrition, School of Medicine, Jinan University, Guangzhou 510632, China; zhousiyao181@163.com (S.Z.); zhaohanqing1227@163.com (H.Z.); yuxiao0227@126.com (Y.X.); lj13422363294@163.com (J.L.); huangqiaoli0421@163.com (Q.H.); yufang_chang0517@163.com (Y.Z.); gff18779773375@163.com (F.G.); 15090786979@163.com (B.X.); 13823040054@163.com (H.Z.); 2 Health Care Centre for Primary and Secondary School of Zhongshan, Zhongshan 528403, China; sophia_em@126.com (X.H.); hzr3805443852@gmail.com (S.H.)

**Keywords:** breakfast, food preferences, eating behavior, children

## Abstract

**Background:** Skipping breakfast, a prevalent issue among children and adolescents, has been reported to be associated with academic performance and long-term health. However, less attention has been given to the types of breakfast foods consumed. Therefore, our study aims to investigate the association between breakfast variety and dietary knowledge, attitude, and practice (KAP) among preadolescents. **Methods:** The study included 1449 students in grades 4–6 from Zhongshan city, Guangdong province. Data were collected through face-to-face field investigation using a validated questionnaire. The questionnaire encompassed sociodemographic characteristics, as well as dietary KAP. **Results:** Among all participants, 1315 reported consuming breakfast daily. Dietary diversity varied significantly: 8.8% consumed only 1 type of food, 52.9% consumed 2–4 types, and 38.3% consumed ≥5 types. Students who consumed a greater variety of breakfast foods exhibited more favorable dietary and lifestyle patterns. Specifically, those who consumed ≥5 types of food showed statistically significant associations with healthier practices, including reduced intake of sugary beverages and night snacks, stronger adherence to dietary guidelines, more positive attitudes toward improving eating habits, longer sleep durations, increased participation in meal preparation, greater dish variety in meals, and higher engagement in daily physical activity. **Conclusions:** Breakfast variety was associated with KAP, particularly when breakfast types ≥ 5, providing more sufficient and favorable evidence for breakfast consumption.

## 1. Introduction

Dietary habits have a significant impact on overall health and wellbeing [1,2]. The China Dietary Guidelines for School-aged Children (2022) emphasize that dietary recommendations for this group should be based on those for the general population and must also be adapted to reflect current changes in the nutritional status of school-aged children. Breakfast is universally recognized as the most crucial meal of the day [3,4], influencing children’s growth, including height and weight development, as well as metabolic function, with effects potentially extending into adulthood [5,6]. The Dietary Guidelines for Chinese School-aged Children (2022) emphasize the importance of consuming a nutritious breakfast daily. It is recommended that school-aged children incorporate four food groups into their breakfast: cereals and potatoes, vegetables and fruits, meat and eggs, and milk, soy, and nuts. If this is not feasible, at least three of these food groups should be consumed. Breakfast skipping is highly prevalent among school-aged children. A study involving 71,390 students in grades 4–12 from South Australian public schools revealed that 55.0% never skipped breakfast, 17.4% occasionally skipped, 18.0% frequently skipped, and 9.5% always skipped breakfast [7]. A cross-sectional study conducted in China found that 61.5% of students consumed breakfast daily, with higher adherence observed among younger students, urban school students, and those attending academic high school [8].

The habit of skipping breakfast among children and preadolescents can have lasting health consequences, as infrequent breakfast consumption (never or 1–3 times per week) during this developmental stage is associated with obesity [9], stress and depression [10], cardiometabolic risk [11], and other adverse outcomes [12]. A systematic review indicated that children who skipped breakfast had an increased risk of overweight and obesity (AOR = 1.59; 95%CI = 1.33–1.90), with the effect being more pronounced in girls [13], and was also linked to a higher risk of dyslipidemia [14]. A Canadian study found that adolescents who skipped breakfast were more likely to experience psychosomatic symptoms compared to those who consumed breakfast regularly (AOR = 2.55; 95% CI = 1.75–3.82) [15]. Research has demonstrated that breakfast positively influences cognitive functioning, including reaction time and visual-sustained attention [16]. Furthermore, infrequent breakfast consumption during childhood and adolescence is associated with reduced academic performance [17] and lower levels of school engagement [18].

While current research has primarily focused on the frequency of breakfast consumption, equal attention should be given to the diversity of food types consumed during breakfast [19]. A regular breakfast that includes a variety of food items is essential for maintaining a balanced diet in children, providing the necessary nutrients and energy required for their physical growth and cognitive development [20,21]. Therefore, this study aims to examine the diversity of breakfast foods among school-aged children and explore the potential association between breakfast food variety and dietary knowledge, attitude, and practice (KAP). Understanding these associations is critical for designing targeted interventions and educational programs aimed at promoting healthy eating behaviors among primary school students. By elucidating the influence of breakfast diversity on dietary practice, this research seeks to provide valuable insights for educators, parents, and health professionals, ultimately supporting to development of evidence-based strategies to enhance students’ overall health and wellbeing.

## 2. Methods

### 2.1. Participants and Settings

We conducted a cross-sectional survey in April 2021, involving multiple primary schools in Zhongshan city, Guangdong province. To ensure representativeness, three districts—Eastern District, Western District, and Shiqi District—were randomly selected from five urban districts in Zhongshan City based on geographical location. Within each selected district, three primary schools were initially identified, resulting in a total of nine schools. However, considering practical participation conditions and the principle of voluntary cooperation, we ensured that at least one school from each district participated. After completing the selection procedures, five schools ultimately participated in the survey: one from the Eastern District, three from the Western District, and one from the Shiqi District. Stratified cluster sampling was conducted among students in grades 4 to 6 from the five selected schools, stratified by school size. All students within the target grade levels were included for participation. The sample size estimation was performed using the following parameters: a two-sided significance level of α = 0.05, statistical power of 1 − β = 95.0%, an estimated nutrition knowledge awareness rate of *p* = 26.1% among Chinese primary school students, an allowable error margin of *d* = 0.1 × *p* = 0.026, and the sample size calculation formula $n=\frac{Z_{1-\alpha /2}^{2}*pq}{d^{2}}$. The sample size was calculated using PASS 15 software. Considering an anticipated data loss rate of 15.0%, the minimum required sample size was determined to be 1262 participants. Ultimately, a total of 1449 students were enrolled in this study, with sex information based on self-report data.

Study participants were required to meet the following criteria: (1) voluntary participation, (2) the cognitive and practical ability to independently complete the questionnaire, and (3) having undergone the school entrance physical examination. The exclusion criteria were as follows: (1) students with specific dietary requirements due to personal illnesses or other medical reasons, and (2) students who were unable to independently complete the questionnaire due to illness or other related factors. This study was conducted in accordance with the ethical standards outlined in the Declaration of Helsinki and was approved by the Medical Ethics Committee of Jinan University (JNUKY-2023-0043).

### 2.2. Questionnaire Design

Validity analysis: This study utilized a self-designed, non-scaled questionnaire and conducted validity analysis through content validity procedures. The “Nutrition Knowledge Questionnaire” and the “Children’s Dietary Attitude and Behavior Questionnaire” (Table 1 and Table 2) were developed based on the survey objectives and by referencing relevant studies conducted in other regions of China. To assess preliminary applicability, a pilot survey was administered to 45 students from Zhongshan Experimental Primary School. Revisions to the questionnaire content and question order were made according to the pilot survey results, student feedback, and expert opinions from the fields of nutrition and child health.

Reliability analysis: The reliability of the questionnaire was assessed using test–retest reliability. A total of 45 fifth-grade students from the Lanbowan School of Zhongshan Experimental Primary School were randomly selected as participants for reliability testing, and the same group was retested two weeks later. The results from the two administrations were analyzed using Pearson correlation coefficient analysis, with a correlation coefficient of r = 0.848 (*p* < 0.01).

### 2.3. Assessments

Data collection was conducted through face-to-face field investigations using a validated questionnaire. The questionnaire was completed by the students themselves, with guidance from teachers and investigators. Information on sociodemographic characteristics was collected, including gender, grade, the presence of a decayed tooth, only-child status within the family, and parental education level. Other variables from the questionnaire were categorized into three domains: dietary knowledge, attitude, and practice. Nutritional knowledge was assessed by assigning 1 point for each correct answer and 0 for incorrect or unknown responses. The total knowledge score ranged from 0 to 20, with higher scores reflecting better nutrition understanding (Table 1). Detailed items of the nutritional attitude and practice are presented in Table 2.

Data on height and weight were obtained from school physical examination records. Body mass index (BMI) was calculated by dividing weight in kilograms by height in meters squared. The BMI z-score (BAZ) was computed using the World Health Organization (WHO) growth reference data for individuals aged 5–19 years [22]. BAZ serves as a standardized indicator of BMI, adjusted for age and sex relative to a reference population. Children’s nutritional status was classified based on BAZ values as follows: Thinness (−2 < BAZ ≤ −1), Normal weight (−1 < BAZ ≤ +1), Overweight (+1 < BAZ ≤ +2), and Obesity (BAZ > +2).

### 2.4. Statistical Analysis

Statistical analysis was conducted using SPSS version 26.0. Continuous variables with a normal distribution were summarized using the Mean ± standard deviation (SD), and comparisons between groups were performed using Student’s t-test for two groups and one-way ANOVA for more than two groups. Bonferroni correction was applied for post hoc pairwise comparisons within groups. For continuous variables with a skewed distribution, the median (P25, P75) was reported, and group comparisons were carried out using non-parametric rank-sum tests. Categorical variables were expressed as percentages (*n*%) and analyzed using the Chi-square test.

Binary logistic regression analysis was conducted to assess the association between skipping breakfast and dietary KAP, presenting odds ratios (ORs) and 95% confidence intervals (CIs). Subsequently, multiple logistic regression analysis was utilized to assess the association (OR and 95%CIs) between the variety of food types consumed during breakfast and dietary KAP. The independent variable X was ranked according to a hierarchical scale ranging from consistent with healthier nutritional practice to more inclination toward unhealthy attitudes or behaviors (excluding the “Nutrition knowledge score”, which was ranked from low to high). A two-sided *p*-value ≤ 0.05 was considered statistically significant.

## 3. Results

### 3.1. Baseline Characteristics and KAP of the Participants

The baseline characteristics, knowledge scores, attitudes, and health-related practices of all students are summarized in Table 3 A total of 1449 students participated in the study, with 1315 (90.8%) reporting daily breakfast consumption over the past week, while 134 (9.2%) skipped breakfast at least once during that period. Among the age groups, 11-year-old students had the highest proportion of daily breakfast consumption, with a statistically significant difference (χ^2^ = 12.89, *p* = 0.005). Similar trends were also observed in different grades. Students in sixth grade exhibited a significantly higher rate of breakfast skipping compared to those in other grades (χ^2^ = 17.45, *p* < 0.001). No significant differences were observed between the two groups regarding gender, nutritional status, being an only child, or parental educational level (Table 3).

Individuals who consumed breakfast daily demonstrated significantly higher willingness to try non-preferred foods (χ^2^ = 15.13, *p* < 0.001), actively participate in dietary activities (χ^2^ = 21.26, *p* < 0.001), pursue nutritionally adequate diets (χ^2^ = 7.37, *p* = 0.025), and modify unhealthy eating habits (χ^2^ = 25.54, *p* < 0.001) compared to those who skipped breakfast. A significantly higher proportion of students who consumed breakfast daily chose ≥5 types of breakfast foods compared to those who did not eat breakfast regularly (χ^2^ = 9.61, *p* = 0.008). Students who adhered to daily breakfast consumption exhibited a stronger preference for water over alternatives such as tea, sugared beverages, and milk (χ^2^ = 26.66, *p* < 0.001). Additionally, these students showed significantly higher consumption frequencies of eggs (≥1 per day) (χ^2^ = 6.92, *p* = 0.009), fruits (≥400 g/d) (χ^2^ = 6.51, *p* = 0.039), nuts (≥1 time/w) (χ^2^ = 14.89, *p* = 0.001), and vegetables (≥5 types/d) (χ^2^ = 16.35, *p* < 0.001). Conversely, the consumption frequencies of staple foods (4–7 times/w) (χ^2^ = 11.70, *p* < 0.001), sugared beverages (≥3 times/w) (χ^2^ = 3.89, *p* = 0.049), and night snacks (≥3 times/w) (χ^2^ = 15.46, *p* < 0.001) were significantly lower among students who regularly consumed breakfast (Table 4).

In terms of dietary practice, individuals who consumed breakfast daily showed a greater likelihood of participating in food preparation with their parents on a daily basis (χ^2^ = 12.50, *p* = 0.006), engaging in independent cooking (≥3 times per week) (χ^2^ = 13.13, *p* = 0.004), and checking expiration dates when purchasing food (every time) (χ^2^ = 22.59, *p* < 0.001). With regard to physical activities, students who consumed breakfast daily demonstrated a significantly higher tendency to engage in vigorous physical activity (daily) (χ^2^ = 10.61, *p* = 0.005) and to be physically active during school breaks (χ^2^ = 5.94, *p* = 0.015) (Table 5).

### 3.2. The Relationship Between Breakfast Frequency and Dietary KAP

**Figure 1:**
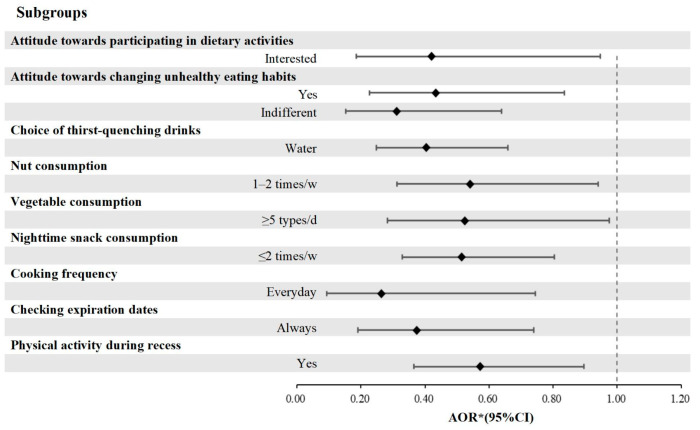
Frequency of breakfast and its significant associations with dietary KAP among all students. * Adjusted by gender, grade, nutritional status, only children, and parental education. Abbreviation: KAP = knowledge, attitude, and practice, AOR = adjusted odds ratio, CI = confidence interval.

After adjusting for grade, gender, nutritional status, being an only child, and parental education level, the associations between breakfast consumption and dietary KAP among all students are presented in Figure 1 and Table 6.

Students with a higher level of interest in dietary activities were more likely to consume breakfast daily compared to those with lower interest (AOR = 0.420, 95%CI = 0.186–0.948). Furthermore, students who expressed either neutral or high levels of interest in modifying unhealthy eating habits exhibited a greater tendency toward regular breakfast consumption than those who showed no interest (AOR = 0.434, 95%CI = 0.226–0.835 & AOR = 0.312, 95%CI = 0.152–0.639, respectively). In addition, students who selected water as their preferred beverage demonstrated a significantly higher likelihood of consuming breakfast regularly compared to those who consumed tea, sugared beverages, or milk (AOR = 0.404, 95%CI = 0.248–0.659). Moreover, students who consumed nuts 1–2 times per week were more likely to eat breakfast daily than those who never consumed nuts (AOR = 0.541, 95%CI = 0.312–0.940). Similarly, students who consumed vegetable ≥5 types/day were more inclined to eat breakfast daily compared to those who consumed only 1–2 types/day (AOR = 0.525, 95%CI = 0.283–0.975). Regarding night snack consumption, students who indulged ≤2 times per week tended to consume breakfast daily than those who consumed night snacks ≥3 times per week (AOR = 0.514, 95%CI = 0.329–0.804).

In terms of culinary skill, students who engaged in independent cooking on a daily basis or consistently checked expiration dates when purchasing food showed a higher likelihood of consuming breakfast daily (AOR = 0.263, 95%CI = 0.093–0.745 &AOR = 0.375, 95%CI = 0.190–0.740, respectively). With regard to physical activity, students who participated in physical activity during class breaks were more inclined to consume breakfast daily compared to their inactive counterparts (AOR = 0.572, 95%CI = 0.365–0.896).

### 3.3. Discrepancy of Breakfast Types Among Different Demographic Characteristics and Dietary KAP Groups

As shown in Table 7, among the 1449 participants in our study, a total of 135 students (9.3%) reported consuming ≤1 type of breakfast, while 776 students (53.6%) consumed 2–4 types of foods for breakfast; furthermore, 538 students (37.1%) consumed ≥5 types within the past seven days. Age and grade distribution revealed significant variation (χ^2^ = 19.76, *p* = 0.003 & χ^2^ = 27.87, *p* < 0.001). In terms of age distribution, children of different ages mainly consume 2–4 types of breakfast, and when divided by grade, the proportion of sixth graders who consume ≤5 types of breakfast is the highest.

Significant differences in nutritional attitudes were observed in the ≥5 breakfast type group, including a higher willingness to try non-preferred foods (χ^2^ = 47.05, *p* < 0.001), greater interest in dietary activities (χ^2^ = 83.69, *p* < 0.001), increased motivation for the diet to meet nutritional requirements (χ^2^ = 35.49, *p* < 0.001), and a more positive attitude toward changing poor eating habits (χ^2^ = 41.28, *p* < 0.001) (Table 8).

Significant differences were observed in nutritional practices, including choices of thirst-quenching drinks (χ^2^ = 7.38, *p* = 0.025), daily water intake (χ^2^ = 73.52, *p* < 0.001), and consumption of milk (χ^2^ = 24.42, *p* < 0.001), eggs (χ^2^ = 55.42, *p* < 0.001), fruits (χ^2^ = 46.79, *p* < 0.001), nuts (χ^2^ = 52.22, *p* < 0.001), and vegetables (χ^2^ = 28.32, *p* < 0.001). The group consuming ≥5 types of breakfast items more frequently met recommended intake levels, with 61.7% of students consuming ≥1100 mL of water daily. Chi-square tests also revealed significant differences in the consumption frequencies of staple foods (χ^2^ = 42.03, *p* < 0.001), meat (χ^2^ = 26.19, *p* < 0.001), sugared beverages (χ^2^ = 22.92, *p* < 0.001), Western fast food (χ^2^ = 14.15, *p* < 0.001), high-calorie snacks (χ^2^ = 8.78, *p* = 0.012), and night snacks (χ^2^ = 13.33, *p* = 0.001) across breakfast type groups, with the high-diversity breakfast group (≥5 types) consistently showing reduced high-frequency consumption of less healthy options (Table 9).

Moreover, this group exhibited significantly higher levels of engagement in various household food-related activities, including participating in food preparation with their parents (χ^2^ = 66.72, *p* < 0.001), cooking a greater variety of dishes (χ^2^ = 44.74, *p* < 0.001), engaging in independent cooking (χ^2^ = 38.64, *p* < 0.001) and checking food expiration dates (χ^2^ = 51.43, *p* < 0.001). Physical activity and sleep patterns further highlighted disparities: high-intensity exercise (χ^2^ = 65.71, *p* < 0.001), ≥2 h of daily physical activity (χ^2^ = 60.74, *p* < 0.001) and ≥9 h of sleep per night (χ^2^ = 62.48, *p* < 0.001) were more prevalent in the group consuming ≥5 types of breakfast items (Table 10).

### 3.4. Types of Breakfast Foods and Their Associations with KAP

**Figure 2:**
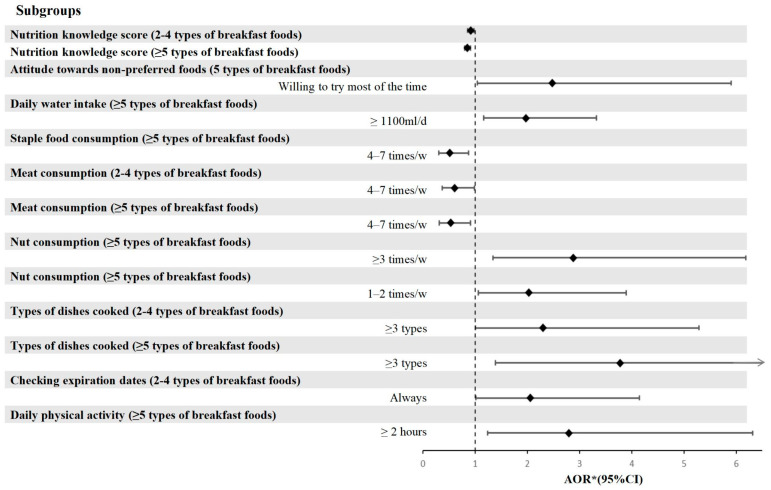
Types of breakfast foods and their significant associations with dietary KAP among all students. * Adjusted by gender, grade, nutritional status, only children, and parental education. Abbreviation: KAP = knowledge, attitude, and practice, AOR = adjusted odds ratio, CI = confidence interval.

After adjusting for gender, grade, nutritional status, being an only child, and parental education level through multivariable analysis, the associations between breakfast food type consumption and dietary KAP among all students are presented in Figure 2 and Table 11.

The scores for nutritional knowledge demonstrated a negative association with an increase in the number of breakfast types consumed. Compared with individuals who consumed ≤1 type of breakfast, those who consumed 2–4 types (AOR = 0.917, 95%CI = 0.859–0.979) and ≥5 types (AOR = 0.854, 95%CI = 0.796–0.915) had significantly lower nutritional knowledge scores. Regarding attitudes, students who consumed ≥5 types of breakfast exhibited greater willingness to try non-preferred foods (AOR = 2.476, 95%CI = 1.038–5.905).

Students with water intake ≥1100 mL/d showed a stronger inclination toward consuming a greater variety of breakfast types (≥5 types) compared to those with water intake <1100 mL/d (AOR = 1.971, 95%CI = 1.168–3.327). For students who consume nuts at least 3 times per week, as well as those who consume nuts 1–2 times per week, both groups were more likely to consume breakfast containing at least 5 food items compared to students who never consumed nuts (AOR = 2.879, 95%CI = 1.341–6.181 & AOR = 2.031, 95%CI = 1.061–3.891, respectively). Students who consumed starchy foods 4–7 times weekly were less likely to eat five or more types of breakfast than those who consumed them 1–3 times per week (AOR = 0.515, 95%CI = 0.303–0.875). Similarly, students who consumed meat 4–7 times per week were significantly less likely to have breakfast containing 2–4 items (AOR = 0.607, 95%CI = 0.373–0.985) or ≥5 items (AOR = 0.529, 95%CI = 0.308–0.909) compared to those who consumed meat 1–3 times per week.

Regarding culinary abilities, students who were capable of preparing at least three distinct dishes were more likely to consume either ≥5 or 2–4 types of breakfast items compared to their counterparts lacking such skills (AOR = 2.301, 95%CI = 1.001–5.292 & AOR = 3.775, 95%CI = 1.389–10.258, respectively). Moreover, students who consistently checked expiration dates when purchasing food items were more likely to consume 2–4 distinct types of food for breakfast, compared to those who never checked expiration dates (AOR = 2.054, 95%CI = 1.017–4.148).

Regarding physical activity, students who engaged in at least 2 h of daily physical activity were more likely to consume five or more types of food for breakfast compared to those who engaged in less than one hour of physical activity (AOR = 2.799, 95%CI = 1.240–6.321).

## 4. Discussion

This study explored the association between breakfast (including frequency and composition) and dietary KAP among schoolchildren in Zhongshan City, Guangdong Province, using a cross-sectional survey design. The results indicated that 38.3% of students who consistently consumed breakfast with at least five food items exhibited healthier dietary attitudes and behaviors, including greater openness to trying new foods, reduced consumption of sugary beverages and night snacks, increased intake of vegetables and nuts, and more active physical activity habits. These findings not only underscore the importance of breakfast quality in shaping children’s health behaviors but also provide empirical support for optimizing breakfast-related intervention strategies.

Our findings reveal that, similar to breakfast frequency, the type of breakfast consumed is uniquely associated with grade level as a demographic characteristic. This may be interpreted as an increase in teenagers’ awareness of healthy breakfast habits as they progress through their school years. With respect to dietary behavior characteristics, a statistically significant difference was observed between breakfast consumption patterns and food type selections. Specifically, children who regularly consume breakfast exhibit significantly higher rates of consuming ≥5 types of breakfast items compared to those who eat breakfast irregularly. This suggests that consistent breakfast consumers tend to adopt more diversified dietary patterns. Notably, breakfast variety demonstrated a negative correlation with nutritional knowledge scores, and this inverse relationship became stronger as food diversity increased—a finding that contradicts the existing literature [23]. Therefore, given the potential discrepancy between theoretical understanding and practical implementation, future research should consider incorporating interventions that complement theoretical nutritional education with practical guidance on implementation.

In terms of dietary attitude characteristics, individuals who consumed a greater variety of breakfast items consistently exhibited more proactive dietary attitudes. Consistent with previous findings, children’s positive attitudes toward breakfast were significantly associated with higher levels of healthy eating self-efficacy (e.g., confidence in selecting nutritious foods) [24]. Increased breakfast diversity was correlated with greater willingness to try disliked foods, enhanced interest in food-related activities, a stronger commitment to nutritional balance, and increased motivation to change unhealthy eating habits. Following adjustment for potential confounders, frequent willingness to try disliked foods was found to be positively associated with daily consumption of ≥5 breakfast varieties. These findings suggest that fostering healthy dietary attitudes in children may enhance the overall quality of their breakfast intake [25]. Existing research confirms that children’s dietary perceptions, including beliefs, attitudes, facilitators, and perceived barriers, serve as a key determinant of breakfast behaviors [26]. Therefore, during the development of a nutritionally balanced breakfast routine, individuals’ cognitive attitudes function as a central regulatory mechanism in dietary decision-making processes [27].

In the realm of nutritional practice, our research revealed that individuals who consumed a diverse range of breakfast items were more frequently engaged in health-promoting behaviors, such as prioritizing water intake and increasing consumption of dairy products, eggs, fruits, vegetables, and nuts. These findings are consistent with previous studies that have demonstrated higher intakes of vegetables and dairy among regular breakfast consumers [8,28,29,30]. Our analysis of nut consumption revealed significantly higher intake in the group consuming ≥5 breakfast varieties compared to those consuming fewer varieties, although overall nut consumption remained below recommended levels across all groups. Adjusted analyses indicated that a greater frequency of nut consumption (1–2 times/week and ≥3 times/week) was positively correlated with consuming ≥5 breakfast varieties, with the strongest associations observed at the highest levels of nut intake. These results underscore the importance of targeted guidance on nut consumption, particularly for individuals who consume 2–4 breakfast varieties [31,32]. With regard to unhealthy diet patterns, individuals with more diverse breakfasts showed significantly lower consumption of Western-style fast food and high-calorie snacks [33]. Although the initial analysis suggested an association between weekly sugar-sweetened beverage intake (≤2 times/week) and both 2–4 and ≥5 breakfast variety groups [34], this relationship was no longer significant after adjusting for confounding variables, potentially due to the uneven distribution of adjusted variables across groups.

However, increased breakfast diversity was associated with lower weekly consumption frequencies of staple foods (4–7 times/week) and meats (4–7 times/week). Following the adjustment of confounding variables, both staple food and meat consumption showed negative correlations with the consumption of ≥5 breakfast varieties. Students who consumed only a single type of breakfast food were more likely to rely exclusively on staple foods or meats, which are perceived as more satiating and convenient. This pattern may have contributed to a habitual preference for these food groups in daily dietary intake. Another study reported a significant relationship between dietary diversity and staple food intake, where lower dietary diversity was associated with higher consumption of staple foods—findings that align with those of our study [35]. Furthermore, students who consume a greater variety of breakfast items tend to prefer high-protein and fiber-rich foods such as vegetables and fruits [36], leading to a reduction in overall intake of carbohydrates and fats. These results are consistent with findings from other studies [37,38].

Regarding culinary practice characteristics, the highest proportion of individuals consuming ≥5 breakfast varieties was observed among those who prepare cooking ingredients with their parents daily, are capable of cooking at least three dishes, regularly engage in independent cooking activities, and routinely check the expiration dates when purchasing food, with statistically significant differences between groups [39]. Correlation analysis revealed that having ≥3 cooking skills was positively associated with the consumption of both 2–4 and ≥5 breakfast varieties, with the strength of association increasing as breakfast diversity increased. Aligning with our findings, a health literacy intervention study targeting adolescent students—including a component focused on cooking skills—demonstrated that improved dietary literacy can lead to positive changes in dietary behavior [40]. Therefore, we recommend enhancing breakfast quality through collaborative efforts between families and schools in a culinary education program.

Regarding physical activity and sleep characteristics, individuals who consumed ≥5 breakfast varieties exhibited the highest rates of participation in high-intensity exercise, engagement in ≥2 h of daily physical activity, and ≥9 h of nightly sleep. After adjustment for potential confounders, engaging in ≥2 h of daily exercise remained positively correlated with consuming ≥5 breakfast varieties. Existing evidence indicates that physical activity may indirectly influence breakfast behaviors by improving overall health, which in turn encourages families to prioritize nutritious breakfast options [34]. Moreover, physical exercise may also indirectly promote breakfast intake by increasing a child’s energy requirements, particularly in the morning when substantial energy is needed to support physical activities [41]. Therefore, our study identified significant associations between healthy lifestyle habits and both the regularity and variety of breakfast, which are reflected through positive dietary attitudes, balanced nutrition, and active lifestyles. The observed association between breakfast patterns and the KAP model provides empirical support for comprehensive public health strategies. Furthermore, several existing intervention studies align with this perspective. For instance, a study conducted among primary school children suggested that a three-month intervention in a healthy school canteen environment can effectively improve children’s eating behaviors [42]. However, prior studies have not specifically addressed interventions targeting the composition or variety of breakfast itself. Therefore, our findings provide foundational insights that may inform the design and implementation of future intervention trials.

## 5. Strengths and Limitations

The strength of this study lies in its incorporation of breakfast diversity—including both consumption frequency and typological variations—into the KAP analytical framework, providing novel insights into children’s dietary behaviors. Secondly, the utilization of a large-scale sample enhanced the representativeness and generalizability of the findings to the broader study area. Thirdly, the comprehensive examination of multiple dimensions of dietary KAP patterns provides a holistic understanding of how the children’s KAP components interact with their breakfast habits. This multidimensional approach establishes a robust scientific foundation for the development of evidence-based interventions targeting childhood dietary behaviors.

This study also has several limitations. First, potential recall bias may exist as the data were self-reported based on participants’ memories. To mitigate this limitation, investigators underwent systematic training and adhered to standardized protocols during data collection, aiming to minimize inconsistencies in participants’ recall. Second, the study focused on students in Grades 4 to 6 from Zhongshan City, which limits the generalizability of the findings. Regional socioeconomic disparities—particularly between urban and rural or less-developed areas—further constrain the applicability of the results beyond the studied population. Finally, the indicators used in this study were mainly qualitative and did not include a detailed assessment of specific breakfast or overall dietary intake. Future research could adopt quantitative dietary assessment methods, such as food frequency questionnaires or 24 h dietary recalls, to enhance the accuracy and reliability of the findings.

## 6. Conclusions

In conclusion, this study demonstrates significant associations between breakfast diversity and health-related behaviors among school-aged children, underscoring breakfast quality as a modifiable factor that can be targeted to improve children’s health behaviors. These findings provide a valuable foundation for the development of future interventions aimed at promoting healthy child development.

## Figures and Tables

**Table 1:** Questions regarding each item in the nutritional knowledge section.

Items
K1: Which nutrient, besides carbohydrate and fat, serves a source of energy?
K2: Which of the following foods is a primary source of carbohydrates?
K3: What proportion of daily energy intake should be provided by breakfast for school-aged children?
K4: What is the function of dietary fiber?
K5: Which of the following foods is rich in dietary fiber?
K6: Among animal-based food, which has the highest nutritional value?
K7: What is the recommended daily limit for salt consumption?
K8: Which of the following foods is a source of high-quality protein?
K9: Which dairy products is considered the most nutritious?
K10: Are trans fatty acids harmful to human body?
K11: Which nutrient in dairy products contribute to bone strength?
K12: What type of food can help prevent iron-deficiency anemia?
K13: Which vitamin is beneficial for the healing of oral ulcers?
K14: Can fruit juice fully replace fresh fruits in the diet?
K15: Which vitamin in cereal grains helps regulate the nervous system?
K16: Is soybean protein considered a high-quality protein?
K17: For school-aged children, what is the recommended daily limit for added sugar intake?
K18: What is the recommended duration of daily physical activity for school-aged children?
K19: Are you familiar with nutrition labels on food packages?
K20: Do you believe that eating healthy meals is important for overall health?

**Table 2:** Detailed questionnaire items related to nutritional attitudes and practices.

Nutritional Attitude	
Attitude towards non-preferred foods	Are you willing to try foods that are not your favorite? (Willing to try most of the time, Willing to try occasionally, Always unwilling to try)
Sources of dietary knowledge	What are your sources of dietary knowledge? (Multiple choice) (Television/Internet, School clubs/Large scale events, Newspapers/Magazines/Books, Classroom instruction, Home, Other)
Dietary activities participation	Has the school or class ever organized any of the following dietary-related activities? (Multiple choice) (Class meetings focused on diet, Specialized dietary education courses, Integration of dietary knowledge into other subjects, Series of informative dietary lectures, Cooking activities, Dietary knowledge competition, Other, None of the above apply)
Attitude towards participating in dietary activities	Are you inclined to participate in the dietary activities organized by your school? (Interested, Neutral, Uninterested)
Expectation of nutritional adequacy	Do you expect your diet to meet your nutritional requirements? (Yes, No, Indifferent)
Attitude towards changing unhealthy eating habits	Do you want to change your unhealthy eating habits? (Yes, No, Indifferent)
Parental dietary expectation	What dietary expectations do your parentes have for you? (Eating whatever I like, Not picky, Not picky and being informed about the nutritional content of different foods)
Perception of body shape	How would you describe your body shape? (Normal, Thin, Obese)
Satisfaction with body shape	Are you satisfied with your body shape? (Very satisfied, Satisfied, Neutral, Not satisfied, Very dissatisfied)
Perception of obesity	What is your opinion on obesity? (Obesity is positively correlated with health, Obesity is negatively correlated with health, Obesity has no correlation with health)
Perceived causes of obesity	Which factors do you believe contribute to obesity? (Multiple choice) (Genetics, Excessive energy intake, Insufficient physical activity, Picky eating, Irregular eating patterns)
Nutritional practice	
Frequency of breakfast	During the past week, how often did you have breakfast? (Everyday, 3–6 times/w, 1–2 times/w, Never)
Reasons for skipping breakfast	During the past week, what were the reasons for not having breakfast? (Applicable only to students who did not select ‘everyday’ in the previous question) (Lack of time, Fullness or loss of appetite, No breakfast preparation available, Dietary considerations for weight loss)
Types of breakfast foods	Which of the following food categories were included in your breakfast on each occasion? (Multiple choices) (Grains, Vegetables, Fruits, Meats, Eggs, Beans, Milk, Nuts)
Choice of thirst-quenching drinks	During the past week, when you were thirsty, which of the following drinks did you choose most often? (Water, Tea, Cola/Sprite/Juice/Milk tea or other sweetened beverages, Milk)
Daily water intake	During the past week, what was your average daily consumption of water(including puried or mineral water)? (≥1100 mL, 600–1000 mL, 200–600 mL, ≤200 mL)
Milk consumption	During the past week, how often did you consume milk or milk products? (≥2 bottles/d, 1 bottle/d, 1 bottle every 1–2 days, 1 bottle of every 3–5 days, 1 bottle of every 6–7 days, never) (each bottle ≈ 250 mL)
Egg consumption	During the past week, how often did you eat eggs? (≥2 times/d, 1 time/d, 3–6 times/w, 1–2 times/w, Never)
Staple food consumption	During the past week, how often did you eat staple foods (such as cereals and potatoes)? (3 times/d, 2 times/d, 1 time/d, 3–5 times/w, 1–2 times/w, Never)
Meat consumption	During the past week, how often did you eat meat (e.g., pork, beef, lamb, chicken, duck, fish, etc.)? (3 times/d, 2 times/d, 1 time/d, 3–5 times/w, 1–2 times/w, Never)
Bean product consumption	During the past week, how often did you consume beans and products (e.g., tofu, soybean milk, beancurd sheet, etc.)? (3 times/d, 2 times/d, 1 time/d, 3–6 times/w, 1–2 times/w, Never)
Fruit consumption	During the past week, how much fruit did you eat per day on average (The edible portion of a medium apple is about 200 g)? (≥400 g/d, 300–400 g/d, 200–300 g/d, 100–200 g/d, <100 g/d)
Nut consumption	During the past week, how often did you eat nuts (e.g., seeds, almonds, walnuts, etc.)? (Everyday, 3–6 times/w, 1–2 times/w, Never)
Vegetable consumption	During the past week, how many different types of vegetables did you eat per day? (≥5 types/d, 3–4 types/d, 1–2 types/d)
Sugared beverage consumption	During the past week, how often did you consume sugared beverages (e.g., cola, soda, juice drinks, functional drinks, lactic acid drinks, milk tea, etc.)? (≥2 times/d, 1 time/d, 3–6 times/w, 1–2 times/w, Never)
Amount of sugared beverage intake	During the past week, what was the average amount of each sugared beverage consumed? (Open-ended question)
Western fast food consumption	During the past week, how often did you eat Western fast food (e.g., KFC/McDonald’s/Pizza Hut, etc.) or take-out meals? (≥2 times/d, 1 time/d, 3–6 times/w, 1–2 times/w, Never)
High-calorie snack consumption	During the past week, how often did you eat high-calorie snacks (e.g., chips, candy, jellies, cookies, spicy bars, puffed foods, etc.)? (≥2 times/d, 1 time/d, 3–6 times/w, 1–2 times/w, Never)
Nighttime snack consumption	During the past week, how often did you eat nighttime snacks? (1 time/d, 3–6 times/w, 1–2 times/w, Never)
Diet-related practice	
Help with food preparation	During the past week, how often did you assist in food preparation tasks (e.g., shopping, washing ingredients, setting utensils, etc.) while your family was cooking? (Everyday, 3–6 times/w, 1–2 times/w, Never)
Types of dishes cooked	How many different kinds of dishes can you prepare independently? (None, Limited to rice only, 1–2 types, 3–4 types, 5–9 types, ≥10 types)
Cooking criteria ranking	Based on your experience, please rank the following factors according to their importance when cooking a dish: Degree of liking, Color appearance, Flavor and taste, Availability of ingredients, Nutritional value
Cooking frequency	How often do you participate in cooking activities, such as preparing rice or cooking dishes, either independently or by assisting your family at home? (Everyday, 3–6 times/w, 1–2 times/w, Never)
Checking expiration dates	When purchasing food at the supermarket, do you check the expiration dates? (Always check the date and packaging carefully, Occasionally check the date and packaging, Never check, Not applicable due to no personal purchases)
Physical activity	
Vigorous physical activity	During the past week, on how many days did you engage in vigorous physical activity (e.g., basketball, soccer, running, swimming, fast biking, brisk dancing, etc.) for more than 60 min, resulting in sweating and increased breathing? (7 d, 6 d, 5 d, 4 d, 3 d, 2 d, 1 d, 0 d)
Daily physical activity	During the past week, how much time did you spend on physical activity per day, including recess, PE classes, extracurricular sports, and commuting to and from school? (≥2 h, 1–2 h, 0.5–1 h, ≤0.5 h)
Physical activity during recess	During the past week, how did you typically spend your recess time? (Engaged in reading or homework, Sat quietly or chatted with classmates, Played with classmates inside the classroom, Engaged in outdoor activities)
Daily sleep duration	During the past week, what was your average daily sleep duration, including naps? (≥9 h, 7–8 h, ≤7 h)

**Table 3:** Comparison of sociodemographic characteristics between individuals who skip breakfast and those who consume breakfast daily.

Variables	Total	Eating Daily	Skipping Breakfast	χ^2^ (t)	*p*
	(*n* = 1449)	(*n* = 1315)	(*n* = 134)		
Demographic characteristics					
Sex, *n* (%)				0.12	0.73
Male	780 (53.83)	706 (53.69)	74 (55.22)		
Female	669 (46.17)	609 (46.31)	60 (44.78)		
Age, *n* (%)				12.89	0.005 *
Ninth	298 (20.57)	256 (19.47)	42 (31.34)		
Tenth	519 (35.82)	470 (35.74)	49 (36.57)		
Eleventh	463 (31.95)	432 (32.85)	31 (23.13)		
Twelfth	169 (11.66)	157 (11.94)	12 (8.96)		
Grade, *n* (%)				17.45	<0.001 *
Fourth	532 (36.71)	461 (35.06)	71 (52.99)		
Fifth	562 (38.79)	520 (39.54)	42 (31.34)		
Sixth	355 (24.50)	334 (25.40)	21 (15.67)		
Nutritional status, *n* (%)				2.29	0.52
Thin	160 (11.04)	143 (10.87)	17 (12.69)		
Normal	867 (59.83)	782 (59.47)	85 (63.43)		
Overweight	180 (12.42)	165 (12.55)	15 (11.19)		
Obesity	242 (16.70)	225 (17.11)	17 (12.69)		
Whether only child, *n* (%)				1.55	0.21
Yes	440 (30.37)	393 (29.89)	47 (35.07)		
No	1009 (69.63)	922 (70.11)	87 (64.93)		
Maternal education, *n* (%)				0.14	0.71
College degree or below	941 (64.94)	852 (64.79)	89 (66.42)		
Bachelor’s degree or above	508 (35.06)	463 (35.21)	45 (33.58)		
Paternal education, *n* (%)				0.41	0.52
College degree or below	838 (57.83)	757 (57.57)	81 (60.45)		
Bachelor’s degree or above	611 (42.17)	558 (42.43)	53 (39.55)		

* Indicates statistical significance (*p* < 0.05).

**Table 4:** Comparison of nutritional knowledge, attitude, and practice between individuals who skip breakfast and those who consume breakfast daily.

Variables	Total	Eating Daily	Skipping Breakfast	χ^2^ (t)	*p*
	(*n* = 1449)	(*n* = 1315)	(*n* = 134)		
Nutrition knowledge score, M (P_25_, P_75_)	13.00 (10.00, 16.00)	13.00 (10.00, 16.00)	13.50 (11.00, 16.00)	1.73	0.08
Nutritional attitude					
Attitude towards non-preferred foods, *n* (%)				15.13	<0.001 *
Willing to try most of the time	584 (40.30)	550 (41.83)	34 (25.37)		
Willing to try occasionally	756 (52.17)	672 (51.10)	84 (62.69)		
Always unwilling to try	109 (7.52)	93 (7.07)	16 (11.94)		
Attitude towards participating in dietary activities, *n* (%)				21.26	<0.001 *
Interested	629 (48.87)	592 (50.47)	37 (32.46)		
Neutral	578 (44.91)	517 (44.08)	61 (53.51)		
Uninterested	80 (6.22)	64 (5.46)	16 (14.04)		
Expectation of nutritional adequacy, *n* (%)				7.37	0.025 *
Yes	1036 (71.50)	951 (72.32)	85 (63.43)		
Indifferent	204 (14.08)	175 (13.31)	29 (21.64)		
No	209 (14.42)	189 (14.37)	20 (14.93)		
Attitude towards changing unhealthy eating habits, *n* (%)				25.54	<0.001 *
Yes	925 (63.84)	856 (65.10)	69 (51.49)		
Indifferent	291 (20.08)	268 (20.38)	23 (17.16)		
No	233 (16.08)	191 (14.52)	42 (31.34)		
Satisfaction with body shape, *n* (%)				3.21	0.20
Satisfied	833 (57.49)	747 (56.81)	86 (64.18)		
Neutral	459 (31.68)	421 (32.02)	38 (28.36)		
Unsatisfied	157 (10.84)	147 (11.18)	10 (7.46)		
Nutritional practice (over the past week)					
Types of breakfast foods, *n* (%)				9.61	0.008 *
≥5 types/d	538 (37.13)	503 (38.25)	35 (26.12)		
2–4 types/d	776 (53.55)	696 (52.93)	80 (59.70)		
≤1 type/d	135 (9.32)	116 (8.82)	19 (14.18)		
Choice of thirst-quenching drinks, *n* (%)				26.66	<0.001 *
Water	1204 (83.09)	1114 (84.71)	90 (67.16)		
Tea, sugared beverages, milk, etc.	245 (16.91)	201 (15.29)	44 (32.84)		
Daily water intake, *n* (%)				2.61	0.11
≥1100 mL/d	691 (47.69)	636 (48.37)	55 (41.04)		
<1100 mL/d	758 (52.31)	679 (51.63)	79 (58.96)		
Milk consumption, *n* (%)				3.24	0.07
≥1 bottle/d	956 (65.98)	877 (66.69)	79 (58.96)		
<1 bottle/d	493 (34.02)	438 (33.31)	55 (41.04)		
Egg consumption, *n* (%)				6.92	0.009 *
≥1 per day	363 (25.05)	342 (26.01)	21 (15.67)		
<1 per day	1086 (74.95)	973 (73.99)	113 (84.33)		
Staple food consumption, *n* (%)				11.7	<0.001 *
4–7 times/w	430 (29.68)	373 (28.37)	57 (42.54)		
1–3 times/w	1019 (70.32)	942 (71.63)	77 (57.46)		
Meat consumption, *n* (%)				1.27	0.26
4–7 times/w	343 (23.67)	306 (23.27)	37 (27.61)		
1–3 times/w	1106 (76.33)	1009 (76.73)	97 (72.39)		
Bean products consumption, *n* (%)				0.1	0.75
4–7 times/w	1010 (69.70)	915 (69.58)	95 (70.90)		
1–3 times/w	439 (30.30)	400 (30.42)	39 (29.10)		
Fruit consumption, *n* (%)				6.51	0.04 *
≥400 g/d	415 (28.64)	389 (29.58)	26 (19.40)		
200–400 g/d	779 (53.76)	700 (53.23)	79 (58.96)		
<200 g/d	255 (17.60)	226 (17.19)	29 (21.64)		
Nut consumption, *n* (%)				14.89	0.001 *
≥3 times/w	493 (34.02)	453 (34.45)	40 (29.85)		
1–2 times/w	716 (49.41)	660 (50.19)	56 (41.79)		
Never	240 (16.56)	202 (15.36)	38 (28.36)		
Vegetable consumption, *n* (%)				16.35	<0.001 *
≥5 types/d	539 (37.20)	507 (38.56)	32 (23.88)		
3–4 types/d	667 (46.03)	601 (45.70)	66 (49.25)		
1–2 types/d	243 (16.77)	207 (15.74)	36 (26.87)		
Sugared beverage consumption, *n* (%)				3.89	0.04 *
≤2 times/w	1249 (86.20)	1141 (86.77)	108 (80.60)		
≥3 times/w	200 (13.80)	174 (13.23)	26 (19.40)		
Western fast food consumption, *n* (%)				0.23	0.63
≤2 times/w	1355 (93.51)	1231 (93.61)	124 (92.54)		
≥3 times/w	94 (6.49)	84 (6.39)	10 (7.46)		
High-calorie snack consumption, *n* (%)				1.55	0.21
≤2 times/w	1081 (74.60)	987 (75.06)	94 (70.15)		
≥3 times/w	368 (25.40)	328 (24.94)	40 (29.85)		
Nighttime snack consumption, *n* (%)				15.46	<0.001 *
≤2 times/w	883 (61.96)	821 (63.59)	62 (46.27)		
≥3 times/w	542 (38.04)	470 (36.41)	72 (53.73)		

* Indicates statistical significance (*p* < 0.05).

**Table 5:** Comparison of dietary practices between individuals who skip breakfast and those who consume breakfast daily.

Variables	Total	Eating Daily	Skipping Breakfast	χ^2^	*p*
	(*n* = 1449)	(*n* = 1315)	(*n* = 134)		
Diet-related practice (over the past week)					
Help with food preparation, *n* (%)				12.5	0.006 *
Everyday	501 (34.58)	471 (35.82)	30 (22.39)		
3–6 times/w	400 (27.61)	363 (27.60)	37 (27.61)		
1–2 times/w	423 (29.19)	373 (28.37)	50 (37.31)		
Never	125 (8.63)	108 (8.21)	17 (12.69)		
Types of dishes cooked, *n* (%)				0.73	0.69
≥3 types	907 (62.59)	827 (62.89)	80 (59.70)		
1–2 types	453 (31.26)	409 (31.10)	44 (32.84)		
None	89 (6.14)	79 (6.01)	10 (7.46)		
Cooking frequency, *n* (%)				13.13	0.004 *
Everyday	278 (19.19)	262 (19.92)	16 (11.94)		
3–6 times/w	336 (23.19)	313 (23.80)	23 (17.16)		
1–2 times/w	700 (48.31)	625 (47.53)	75 (55.97)		
Never	135 (9.32)	115 (8.75)	20 (14.93)		
Checking expiration dates, *n* (%)				22.59	<0.001 *
Always	992 (68.46)	923 (70.19)	69 (51.49)		
Occasionally	348 (24.02)	303 (23.04)	45 (33.58)		
Never	109 (7.52)	89 (6.77)	20 (14.93)		
Physical activity (over the past week)					
Vigorous physical activity, *n* (%)				10.61	0.005 *
Everyday	630 (43.48)	585 (44.49)	45 (33.58)		
4–6 days	504 (34.78)	458 (34.83)	46 (34.33)		
1–3 days & Never	315 (21.74)	272 (20.68)	43 (32.09)		
Daily physical activity, *n* (%)				3.6	0.17
≥2 h	425 (29.33)	394 (29.96)	31 (23.13)		
1–2 h	763 (52.66)	690 (52.47)	73 (54.48)		
≤1 h	261 (18.01)	231 (17.57)	30 (22.39)		
Physical activity during recess, *n* (%)				5.94	0.02 *
Yes	740 (51.07)	685 (52.09)	55 (41.04)		
No	709 (48.93)	630 (47.91)	79 (58.96)		
Daily sleep duration, *n* (%)				2.45	0.12
≥9 h	677 (46.72)	623 (47.38)	54 (40.30)		
≤8 h	772 (53.28)	692 (52.62)	80 (59.70)		

* Indicates statistical significance (*p* < 0.05).

**Table 6:** Frequency of breakfast and its associations with dietary KAP among all students.

Variable	Frequency of Breakfast for All Students	
	OR (95%CI)	AOR * (95%CI)
Nutrition knowledge score	1.009 (0.951–1.071)	1.002 (0.943–1.065)
Nutritional attitude		
Attitude towards non-preferred foods		
Willing to try most of the time	0.676 (0.311–1.468)	0.718 (0.327–1.576)
Willing to try occasionally	0.897 (0.452–1.783)	0.938 (0.465–1.892)
Always unwilling to try	Ref.	Ref.
Attitude towards participating in dietary activities		
Interested	0.424 (0.191–0.939)	0.420 (0.186–0.948)
Neutral	0.540 (0.26–1.119)	0.557 (0.265–1.171)
Uninterested	Ref.	Ref.
Expectation of nutritional adequacy		
Yes	1.208 (0.598–2.441)	1.085 (0.525–2.242)
Indifferent	0.858 (0.401–1.837)	0.859 (0.395–1.867)
No	Ref.	Ref.
Attitude towards changing unhealthy eating habits		
Yes	0.439 (0.233–0.825)	0.434 (0.226–0.835)
Indifferent	0.333 (0.166–0.665)	0.312 (0.152–0.639)
No	Ref.	Ref.
Satisfaction with body shape		
Satisfied	1.034 (0.475–2.253)	1.027 (0.464–2.269)
Neutral	0.837 (0.366–1.915)	0.843 (0.363–1.961)
Unsatisfied	Ref.	Ref.
Nutritional practice (over the past week)		
Types of breakfast foods		
≥5 types/d	0.614 (0.289–1.304)	0.559 (0.259–1.205)
2–4 types/d	0.620 (0.316–1.216)	0.528 (0.265–1.052)
≤1 type/d	Ref.	Ref.
Choice of thirst-quenching drinks		
Water	0.420 (0.261–0.675)	0.404 (0.248–0.659)
Others (Tea, sugared beverages, milk, etc.)	Ref.	Ref.
Daily water intake		
≥1100 mL/d	1.172 (0.745–1.846)	1.106 (0.694–1.765)
<1100 mL/d	Ref.	Ref.
Milk consumption		
≥1 bottle/d	1.042 (0.664–1.637)	1.056 (0.668–1.670)
<1 bottle/d	Ref.	Ref.
Egg consumption		
≥1 per day	0.867 (0.493–1.523)	0.836 (0.468–1.492)
<1 per day	Ref.	Ref.
Staple food consumption		
4–7 times/w	1.407 (0.883–2.244)	1.426 (0.885–2.298)
1–3 times/w	Ref.	Ref.
Meat consumption		
4–7 times/w	0.868 (0.516–1.463)	0.870 (0.508–1.488)
1–3 times/w	Ref.	Ref.
Bean products consumption		
4–7 times/w	0.839 (0.514–1.370)	0.853 (0.519–1.404)
1–3 times/w	Ref.	Ref.
Fruit consumption		
≥400 g/d	1.016 (0.495–2.086)	1.106 (0.531–2.306)
200–400 g/d	1.746 (0.975–3.129)	1.762 (0.969–3.201)
<200 g/d	Ref.	Ref.
Nut consumption		
≥3 times/w	0.589 (0.320–1.083)	0.607 (0.326–1.132)
1–2 times/w	0.542 (0.316–0.932)	0.541 (0.312–0.940)
Never	Ref.	Ref.
Vegetable consumption		
≥5 types/d	0.496 (0.270–0.914)	0.525 (0.283–0.975)
3–4 types/d	0.654 (0.381–1.121)	0.657 (0.380–1.136)
1–2 types/d	Ref.	Ref.
Sugared beverage consumption		
≤2 times/w	1.204 (0.649–2.236)	1.197 (0.641–2.236)
≥3 times/w	Ref.	Ref.
Western fast food consumption		
≤2 times/w	1.567 (0.650–3.778)	1.543 (0.630–3.782)
≥3 times/w	Ref.	Ref.
High-calorie snack consumption		
≤2 times/w	0.905 (0.549–1.492)	0.905 (0.544–1.506)
≥3 times/w	Ref.	Ref.
Nighttime snack consumption		
≤2 times/w	0.517 (0.334–0.800)	0.514 (0.329–0.804)
≥3 times/w	Ref.	Ref.
Diet-related practice (over the past week)		
Help with food preparation		
Everyday	1.264 (0.513–3.116)	1.305 (0.514–3.311)
3–6 times/w	1.464 (0.624–3.435)	1.428 (0.593–3.440)
1–2 times/w	1.279 (0.578–2.830)	1.263 (0.560–2.846)
Never	Ref.	Ref.
Types of dishes cooked		
≥3 types	3.271 (1.119–9.563)	2.695 (0.904–8.035)
1–2 types	1.828 (0.637–5.251)	1.609 (0.551–4.700)
None	Ref.	Ref.
Cooking frequency		
Everyday	0.267 (0.096–0.742)	0.263 (0.093–0.745)
3–6 times/w	0.433 (0.172–1.091)	0.444 (0.174–1.134)
1–2 times/w	0.642 (0.292–1.409)	0.629 (0.283–1.394)
Never	Ref.	Ref.
Checking expiration dates		
Always	0.400 (0.206–0.776)	0.375 (0.190–0.740)
Occasionally	0.726 (0.370–1.426)	0.687 (0.344–1.370)
Never	Ref.	Ref.
Physical activity (over the past week)		
Vigorous physical activity		
Everyday	0.782 (0.437–1.401)	0.743 (0.406–1.359)
4–6 days	0.607 (0.347–1.060)	0.623 (0.351–1.108)
1–3 days & Never	Ref.	Ref.
Daily physical activity		
≥2 h	1.006 (0.511–1.981)	1.058 (0.531–2.107)
1–2 h	0.940 (0.546–1.619)	0.933 (0.537–1.622)
≤1 h	Ref.	Ref.
Physical activity during recess		
Yes	0.538 (0.347–0.833)	0.572 (0.365–0.896)
No	Ref.	Ref.
Daily sleep duration		
≥9 h	1.082 (0.670–1.748)	1.106 (0.678–1.806)
≤8 h	Ref.	Ref.

*: Adjusted by gender, grade, nutritional status, only children, and parental education level. KAP = knowledge, attitude, and practice, AOR = adjusted odds ratio, CI = confidence interval.

**Table 7:** Comparison of sociodemographic characteristics among individuals who consume different types of breakfast foods.

Variables	Types of Breakfast Foods			χ^2^ (F)	*p*
	≤1 (*n* = 135)	2–4 (*n* = 776)	≥5 (*n* = 538)		
Sex, *n* (%)				0.62	0.74
Male	77 (57.04)	415 (53.48)	288 (53.53)		
Female	58 (42.96)	361 (46.52)	250 (46.47)		
Age, *n* (%)				19.76	0.003 *
Ninth	15 (11.11)	185 (23.84)	98 (18.22)		
Tenth	58 (42.96)	280 (36.08)	181 (33.64)		
Eleventh	48 (35.56)	224 (28.87)	191 (35.50)		
Twelfth	14 (10.37)	87 (11.21)	68 (12.64)		
Grade, *n* (%)				27.87	<0.001 *
Fourth	35 (25.93)	308 (39.69)	189 (35.13)		
Fifth	70 (51.85)	307 (39.56)	185 (34.39)		
Sixth	30 (22.22)	161 (20.75)	164 (30.48)		
Nutritional status, *n* (%)				7.10	0.31
Thin	10 (7.41)	96 (12.37)	54 (10.04)		
Normal	90 (66.67)	452 (58.25)	325 (60.41)		
Overweight	13 (9.63)	104 (13.40)	63 (11.71)		
Obesity	22 (16.30)	124 (15.98)	96 (17.84)		
Whether only child, *n* (%)				0.66	0.72
Yes	38 (28.15)	242 (31.19)	160 (29.74)		
No	97 (71.85)	534 (68.81)	378 (70.26)		
Maternal education, *n* (%)				5.87	0.05
College degree or below	98 (72.59)	510 (65.72)	333 (61.90)		
Bachelor’s degree or above	37 (27.41)	266 (34.28)	205 (38.10)		
Paternal education, *n* (%)				4.00	0.14
College degree or below	89 (65.93)	443 (57.09)	306 (56.88)		
Bachelor’s degree or above	46 (34.07)	333 (42.91)	232 (43.12)		

* Indicates statistical significance (*p* < 0.05).

**Table 8:** Comparison of nutritional knowledge and attitude among individuals who consume different types of breakfast foods.

Variables	Types of Breakfast Foods			χ^2^ (F)	*p*
	≤1 (*n* = 135)	2–4 (*n* = 776)	≥5 (*n* = 538)		
Nutrition knowledge score, M (P_25_, P_75_)	14.00 (11.00, 17.00)	13.00 (11.00, 16.00)	12.00 (10.00, 15.00)	24.75 ^#^	<0.001 *
Nutritional attitude					
Attitude towards non-preferred foods, *n* (%)				47.05	<0.001 *
Willing to try most of the time	40 (29.63)	270 (34.79)	274 (50.93)		
Willing to try occasionally	77 (57.04)	441 (56.83)	238 (44.24)		
Always unwilling to try	18 (13.33)	65 (8.38)	26 (4.83)		
Attitude towards participating in dietary activities, *n* (%)				83.69	<0.001 *
Interested	27 (23.89)	286 (42.25)	316 (63.58)		
Neutral	77 (68.14)	341 (50.37)	160 (32.19)		
Uninterested	9 (7.96)	50 (7.39)	21 (4.23)		
Expectation of nutritional adequacy, *n* (%)				35.49	<0.001 *
Yes	80 (59.26)	525 (67.65)	431 (80.11)		
Indifferent	28 (20.74)	126 (16.24)	50 (9.29)		
No	27 (20.00)	125 (16.11)	57 (10.59)		
Attitude towards changing unhealthy eating habits, *n* (%)				41.28	<0.001 *
Yes	64 (47.41)	467 (60.18)	394 (73.23)		
Indifferent	41 (30.37)	173 (22.29)	77 (14.31)		
No	30 (22.22)	136 (17.53)	67 (12.45)		
Satisfaction with body shape, *n* (%)				6.25	0.18
Satisfied	86 (63.70)	450 (57.99)	297 (55.20)		
Neutral	34 (25.19)	252 (32.47)	173 (32.16)		
Unsatisfied	15 (11.11)	74 (9.54)	68 (12.64)		

* Indicates statistical significance (*p* < 0.05). ^#^ Kruskal–Wallis test.

**Table 9:** Comparison of nutritional practices among individuals who consume different types of breakfast foods.

Variables	Types of Breakfast Foods			χ^2^	*p*
	≤1 (*n* = 135)	2–4 (*n* = 776)	≥5 (*n* = 538)		
Nutritional practice (over the past week)					
Choice of thirst-quenching drinks, *n* (%)				7.38	0.03 *
Water	102 (75.56)	643 (82.86)	459 (85.32)		
Tea, sugared beverages, milk, etc.	33 (24.44)	133 (17.14)	79 (14.68)		
Daily water intake, *n* (%)				73.52	<0.001 *
≥1100 mL/d	40 (29.63)	319 (41.11)	332 (61.71)		
<1100 mL/d	95 (70.37)	457 (58.89)	206 (38.29)		
Milk consumption, *n* (%)				24.42	<0.001 *
≥1 bottle/d	82 (60.74)	476 (61.34)	398 (73.98)		
<1 bottle/d	53 (39.26)	300 (38.66)	140 (26.02)		
Egg consumption, *n* (%)				55.42	<0.001 *
≥1 per day	23 (17.04)	146 (18.81)	194 (36.06)		
<1 per day	112 (82.96)	630 (81.19)	344 (63.94)		
Staple food consumption, *n* (%)				42.03	<0.001 *
4–7 times/w	56 (41.48)	267 (34.41)	107 (19.89)		
1–3 times/w	79 (58.52)	509 (65.59)	431 (80.11)		
Meat consumption, *n* (%)				26.19	<0.001 *
4–7 times/w	49 (36.30)	201 (25.90)	93 (17.29)		
1–3 times/w	86 (63.70)	575 (74.10)	445 (82.71)		
Bean products consumption, *n* (%)				3.68	0.16
4–7 times/w	98 (72.59)	553 (71.26)	359 (66.73)		
1–3 times/w	37 (27.41)	223 (28.74)	179 (33.27)		
Fruit consumption, *n* (%)				46.79	<0.001 *
≥400 g/d	29 (21.48)	197 (25.39)	189 (35.13)		
200–400 g/d	71 (52.59)	410 (52.84)	298 (55.39)		
<200 g/d	35 (25.93)	169 (21.78)	51 (9.48)		
Nut consumption, *n* (%)				52.22	<0.001 *
≥3 times/w	35 (25.93)	249 (32.09)	209 (38.85)		
1–2 times/w	60 (44.44)	371 (47.81)	285 (52.97)		
Never	40 (29.63)	156 (20.10)	44 (8.18)		
Vegetable consumption, *n* (%)				28.32	<0.001 *
≥5 types/d	41 (30.37)	273 (35.18)	225 (41.82)		
3–4 types/d	57 (42.22)	356 (45.88)	254 (47.21)		
1–2 types/d	37 (27.41)	147 (18.94)	59 (10.97)		
Sugared beverage consumption, *n* (%)				22.92	<0.001 *
≤2 times/w	99 (73.33)	670 (86.34)	480 (89.22)		
≥3 times/w	36 (26.67)	106 (13.66)	58 (10.78)		
Western fast food consumption, *n* (%)				14.15	<0.001 *
≤2 times/w	116 (85.93)	731 (94.20)	508 (94.42)		
≥3 times/w	19 (14.07)	45 (5.80)	30 (5.58)		
High-calorie snack consumption, *n* (%)				8.78	0.012 *
≤2 times/w	96 (71.11)	560 (72.16)	425 (79.00)		
≥3 times/w	39 (28.89)	216 (27.84)	113 (21.00)		
Nighttime snack consumption, *n* (%)				13.33	0.001 *
≤2 times/w	77 (57.89)	444 (58.42)	362 (68.05)		
≥3 times/w	56 (42.11)	316 (41.58)	170 (31.95)		

* Indicates statistical significance (*p* < 0.05).

**Table 10:** Comparison of dietary practices among individuals who consume different types of breakfast foods.

Variables	Types of Breakfast Foods			χ^2^	*p*
	≤1 (*n* = 135)	2–4 (*n* = 776)	≥5 (*n* = 538)		
Diet-related practice (over the past week)					
Help with food preparation, *n* (%)				66.72	<0.001 *
Everyday	34 (25.19)	217 (27.96)	250 (46.47)		
3–6 times/w	34 (25.19)	224 (28.87)	142 (26.39)		
1–2 times/w	49 (36.30)	253 (32.60)	121 (22.49)		
Never	18 (13.33)	82 (10.57)	25 (4.65)		
Types of dishes cooked, *n* (%)				44.74	<0.001 *
≥3 types	59 (43.70)	468 (60.31)	380 (70.63)		
1–2 types	58 (42.96)	254 (32.73)	141 (26.21)		
None	18 (13.33)	54 (6.96)	17 (3.16)		
Cooking frequency, *n* (%)				38.64	<0.001 *
Eveyday	24 (17.78)	124 (15.98)	130 (24.16)		
3–6 times/w	30 (22.22)	161 (20.75)	145 (26.95)		
1–2 times/w	61 (45.19)	404 (52.06)	235 (43.68)		
Never	20 (14.81)	87 (11.21)	28 (5.20)		
Checking expiration dates, *n* (%)				51.43	<0.001 *
Always	68 (50.37)	503 (64.82)	421 (78.25)		
Occasionally	47 (34.81)	210 (27.06)	91 (16.91)		
Never	20 (14.81)	63 (8.12)	26 (4.83)		
Physical activity (over the past week)					
Vigorous physical activity, *n* (%)				65.71	<0.001 *
Everyday	48 (35.56)	279 (35.95)	303 (56.32)		
4–6 days	46 (34.07)	296 (38.14)	162 (30.11)		
1–3 days & Never	41 (30.37)	201 (25.90)	73 (13.57)		
Daily physical activity, *n* (%)				60.74	<0.001 *
≥2 h	21 (15.56)	196 (25.26)	208 (38.66)		
1–2 h	75 (55.56)	414 (53.35)	274 (50.93)		
≤1 h	39 (28.89)	166 (21.39)	56 (10.41)		
Physical activity during recess, *n* (%)				3.57	0.17
Yes	75 (55.56)	379 (48.84)	286 (53.16)		
No	60 (44.44)	397 (51.16)	252 (46.84)		
Daily sleep duration, *n* (%)				62.48	<0.001 *
≥9 h	43 (31.85)	312 (40.21)	322 (59.85)		
≤8 h	92 (68.15)	464 (59.79)	216 (40.15)		

* Indicates statistical significance (*p* < 0.05).

**Table 11:** Types of breakfast foods and their associations with dietary KAP among all students.

Variable	Types of Breakfast Foods for All Students			
	2 ≤ Types ≤ 4		Types ≥ 5	
	OR (95%CI)	AOR * ( 95%CI)	OR (95%CI)	AOR * (95%CI)
Nutrition knowledge score	0.917 (0.86–0.978)	0.917 (0.859–0.979)	0.855 (0.798–0.916)	0.854 (0.796–0.915)
Nutritional attitude				
Attitude towards non-preferred foods				
Willing to try most of the time	1.589 (0.743–3.397)	1.592 (0.734–3.451)	2.365 (1.004–5.569)	2.476 (1.038–5.905)
Willing to try occasionally	1.499 (0.760–2.956)	1.440 (0.720–2.883)	1.989 (0.906–4.365)	1.974 (0.888–4.390)
Always unwilling to try	Ref.	Ref.	Ref.	Ref.
*p*-trend	0.561	0.516	0.147	0.121
Attitude towards participating in dietary activities				
Interested	1.055 (0.414–2.691)	1.023 (0.392–2.669)	1.418 (0.502–4.004)	1.231 (0.424–3.573)
Neutral	0.568 (0.244–1.320)	0.578 (0.243–1.375)	0.547 (0.211–1.418)	0.510 (0.192–1.360)
Uninterested	Ref.	Ref.	Ref.	Ref.
*p*-trend	0.230	0.209	0.024	0.030
Expectation of nutritional adequacy				
Yes	0.863 (0.408–1.825)	0.823 (0.381–1.775)	0.831 (0.357–1.933)	0.835 (0.351–1.986)
Indifferent	1.083 (0.519–2.260)	1.044 (0.496–2.197)	1.068 (0.457–2.496)	0.996 (0.422–2.349)
No	Ref.	Ref.	Ref.	Ref.
*p*-trend	0.682	0.632	0.687	0.693
Attitude towards changing unhealthy eating habits				
Yes	1.054 (0.505–2.199)	1.104 (0.522–2.335)	1.022 (0.457–2.283)	1.065 (0.469–2.417)
Indifferent	0.630 (0.321–1.234)	0.661 (0.335–1.304)	0.612 (0.285–1.316)	0.663 (0.306–1.437)
No	Ref.	Ref.	Ref.	Ref.
*p*-trend	0.642	0.655	0.650	0.621
Satisfaction with body shape				
Satisfied	1.100 (0.529–2.289)	1.019 (0.481–2.158)	0.900 (0.417–1.943)	0.823 (0.374–1.811)
Neutral	1.371 (0.623–3.020)	1.256 (0.559–2.821)	1.071 (0.467–2.460)	0.923 (0.393–2.165)
Unsatisfied	Ref.	Ref.	Ref.	Ref.
*p*-trend	0.852	0.902	0.659	0.741
Nutritional practice (over the past week)				
Choice of thirst-quenching drinks				
Water	1.361 (0.804–2.306)	1.385 (0.809–2.372)	1.182 (0.662–2.110)	1.206 (0.667–2.179)
Others (Tea, sugared beverages, milk, etc.)	Ref.	Ref.	Ref.	Ref.
Daily water intake				
≥1100 mL/d	1.287 (0.788–2.100)	1.252 (0.763–2.054)	1.958 (1.167–3.286)	1.971 (1.168–3.327)
<1100 mL/d	Ref.	Ref.	Ref.	Ref.
Milk consumption				
≥1 bottle/d	0.742 (0.462–1.190)	0.709 (0.439–1.146)	0.911 (0.544–1.526)	0.852 (0.505–1.436)
<1 bottle/d	Ref.	Ref.	Ref.	Ref.
Egg consumption				
≥1 per day	0.946 (0.528–1.695)	0.908 (0.504–1.636)	1.426 (0.779–2.609)	1.474 (0.801–2.710)
<1 per day	Ref.	Ref.	Ref.	Ref.
Staple food consumption				
4–7 times/w	0.862 (0.536–1.386)	0.857 (0.530–1.385)	0.497 (0.294–0.838)	0.515 (0.303–0.875)
1–3 times/w	Ref.	Ref.	Ref.	Ref.
Meat consumption				
4–7 times/w	0.585 (0.362–0.945)	0.607 (0.373–0.985)	0.535 (0.314–0.913)	0.529 (0.308–0.909)
1–3 times/w	Ref.	Ref.	Ref.	Ref.
Bean products consumption				
4–7 times/w	0.971 (0.573–1.645)	0.948 (0.557–1.614)	1.100 (0.63–1.921)	1.026 (0.584–1.801)
1–3 times/w	Ref.	Ref.	Ref.	Ref.
Fruit consumption				
≥400 g/d	1.211 (0.618–2.373)	1.215 (0.616–2.398)	1.858 (0.886–3.898)	1.827 (0.862–3.869)
200–400 g/d	1.121 (0.658–1.910)	1.119 (0.652–1.918)	1.620 (0.876–2.993)	1.700 (0.913–3.167)
<200 g/d	Ref.	Ref.	Ref.	Ref.
Nut consumption				
≥3 times/w	1.801 (0.923–3.517)	1.755 (0.891–3.458)	3.038 (1.428–6.464)	2.879 (1.341–6.181)
1–2 times/w	1.091 (0.634–1.879)	1.098 (0.633–1.904)	2.016 (1.061–3.831)	2.031 (1.061–3.891)
Never	Ref.	Ref.	Ref.	Ref.
*p*-trend	0.067	0.081	0.012	0.018
Vegetable consumption				
≥5 types/d	1.331 (0.715–2.478)	1.328 (0.706–2.497)	1.298 (0.653–2.579)	1.272 (0.634–2.553)
3–4 types/d	1.487 (0.849–2.602)	1.486 (0.839–2.632)	1.669 (0.888–3.139)	1.700 (0.895–3.229)
1–2 types/d	Ref.	Ref.	Ref.	Ref.
*p*-trend	0.574	0.624	0.867	0.976
Sugared beverage consumption				
≤2 times/w	1.881 (1.014–3.489)	1.766 (0.940–3.317)	2.044 (1.034–4.040)	1.822 (0.910–3.647)
≥3 times/w	Ref.	Ref.	Ref.	Ref.
Western fast food consumption				
≤2 times/w	1.619 (0.731–3.585)	1.487 (0.660–3.354)	1.188 (0.495–2.852)	1.114 (0.455–2.724)
≥3 times/w	Ref.	Ref.	Ref.	Ref.
High-calorie snack consumption				
≤2 times/w	0.909 (0.530–1.556)	0.938 (0.544–1.616)	1.168 (0.652–2.094)	1.201 (0.666–2.166)
≥3 times/w	Ref.	Ref.	Ref.	Ref.
Nighttime snack consumption				
≤2 times/w	0.710 (0.444–1.134)	0.723 (0.450–1.163)	0.894 (0.539–1.481)	0.899 (0.539–1.499)
≥3 times/w	Ref.	Ref.	Ref.	Ref.
Diet-related practice (over the past week)				
Help with food preparation				
Everyday	0.767 (0.307–1.918)	0.791 (0.312–2.001)	1.225 (0.429–3.498)	1.137 (0.394–3.283)
3–6 times/w	0.847 (0.348–2.060)	0.886 (0.361–2.173)	0.887 (0.316–2.488)	0.835 (0.295–2.362)
1–2 times/w	0.732 (0.326–1.643)	0.731 (0.323–1.657)	0.788 (0.303–2.052)	0.706 (0.269–1.856)
Never	Ref.	Ref.	Ref.	Ref.
*p*-trend	0.749	0.753	0.259	0.252
Types of dishes cooked				
≥3 types	2.568 (1.138–5.795)	2.301 (1.001–5.292)	3.917 (1.467–10.458)	3.775 (1.389–10.258)
1–2 types	1.323 (0.610–2.871)	1.263 (0.573–2.784)	1.751 (0.675–4.547)	1.741 (0.661–4.588)
None	Ref.	Ref.	Ref.	Ref.
*p*-trend	0.001	0.003	<0.001	<0.001
Cooking frequency				
Everyday	0.603 (0.235–1.547)	0.632 (0.243–1.642)	0.537 (0.183–1.582)	0.615 (0.206–1.837)
3–6 times/w	0.539 (0.217–1.340)	0.561 (0.222–1.417)	0.619 (0.219–1.752)	0.768 (0.266–2.213)
1–2 times/w	1.033 (0.467–2.287)	1.024 (0.457–2.296)	1.041 (0.411–2.636)	1.127 (0.439–2.889)
Never	Ref.	Ref.	Ref.	Ref.
*p*-trend	0.052	0.087	0.048	0.076
Checking expiration dates				
Always	1.946 (0.972–3.897)	2.054 (1.017–4.148)	2.118 (0.931–4.820)	2.145 (0.935–4.919)
Occasionally	1.560 (0.757–3.213)	1.603 (0.770–3.336)	1.674 (0.707–3.968)	1.724 (0.721–4.120)
Never	Ref.	Ref.	Ref.	Ref.
*p*-trend	0.080	0.059	0.089	0.068
Physical activity (over the past week)				
Vigorous physical activity				
Everyday	0.673 (0.375–1.208)	0.644 (0.353–1.173)	0.947 (0.497–1.806)	0.893 (0.460–1.731)
4–6 days	1.221 (0.696–2.143)	1.234 (0.696–2.187)	1.346 (0.715–2.534)	1.266 (0.664–2.414)
1–3 days & Never	Ref.	Ref.	Ref.	Ref.
*p*-trend	0.507	0.479	0.629	0.639
Daily physical activity				
≥2 h	1.944 (0.916–4.125)	1.955 (0.915–4.180)	2.777 (1.241–6.212)	2.799 (1.240–6.321)
1–2 h	1.050 (0.614–1.794)	1.070 (0.622–1.841)	1.305 (0.714–2.387)	1.298 (0.705–2.391)
≤1 h	Ref.	Ref.	Ref.	Ref.
*p*-trend	0.107	0.089	0.011	0.011
Physical activity during recess				
Yes	0.659 (0.422–1.028)	0.675 (0.430–1.059)	0.751 (0.466–1.211)	0.774 (0.477–1.256)
No	Ref.	Ref.	Ref.	Ref.
Daily sleep duration				
≥9 h	1.063 (0.643–1.758)	1.079 (0.649–1.793)	1.477 (0.866–2.519)	1.476 (0.860–2.532)
≤8 h	Ref.	Ref.	Ref.	Ref.

*: Adjusted by gender, grade, nutritional status, only children, and parental education level. KAP = knowledge, attitude, and practice, AOR = adjusted odds ratio, CI = confidence interval.

## Data Availability

All data collected were anonymized and stored securely to protect participant confidentiality. No personally identifiable information was disclosed during data analysis or reporting. The data that support the findings of this study are available on request from the corresponding author (L.W.), upon reasonable request.
